# Myopia: Risk Factors, Disease Mechanisms, Diagnostic Modalities, and Therapeutic Options 2019

**DOI:** 10.1155/2020/5375927

**Published:** 2020-03-07

**Authors:** Magorzata Mrugacz, Marzena Gajecka, Ewa Mrukwa-Kominek, Katarzyna J. Witkowska

**Affiliations:** ^1^Medical University of Bialystok, Bialystok, Poland; ^2^Poznan University of Medical Sciences, Poznań, Poland; ^3^Medical University of Silesia, Katowice, Poland; ^4^Medical University of Vienna, Vienna, Austria

Myopia is a global problem, being particularly prevalent in the urban areas of east and southeast Asia. It is estimated that 2.5 billion people will be affected by myopia within the next decade. In addition to the economic and social burdens, associated ocular complications may lead to visual impairment. Myopia has a diverse etiology, with both environmental and genetic factors believed to be involved in the myopia's development and progression. Genetic linkage studies have mapped the dozen loci, while association studies have found more than 70 different genes. Many of these genes are involved in common biological pathways known to mediate extracellular matrix composition and regulate connective tissue remodelling. Other associated genomic regions suggest novel mechanisms in the etiology of high myopia, such as mitochondrial-mediated cell death and photoreceptor-mediated visual signal transmission. The environmental factors implicated in myopia include near work, light exposure, lack of physical activity, diet, a higher level of education, and urbanization. The interactions between genes and environmental factors may be significant in determining individual risks of high myopia and may help explain the pathogenetic mechanisms of myopia in human population [1, 2].

The first paper of this special issue addresses the review the current evidence for its complex genetics and evaluates the known or candidate genes and loci. In addition, the authors discuss recent investigations regarding the role of environmental factors and current research aimed at elucidating the signaling pathways involved in the pathogenesis of myopia. The second paper presents the study on the outcomes of femtosecond laser-assisted implantation of a 355-degree intracorneal ring (ICR) (Keraring) in patients with keratoconus. The mean sphere, cylinder, and spherical equivalent have been changed dramatically from preoperative to 3 months postoperative, which is statistically significant (*P* ≤ 0.001), and the changes between 1 and 2 years and 2 and 3 years are also considerable and statistically significant. Implantation of a 355-degree intracorneal keraring using femtosecond laser improved the visual, refractive, and topographic parameters in keratoconus patients, with a high rate of ICR extrusion and instability. The authors of the third paper have observed that low-dose atropine does inhibit the short-term effect of hyperopic blur on choroidal thickness and, when used alone, does cause a slight thickening of the choroid in young healthy myopic adults. The three subsequent papers present the prevalence and related factors for myopia in school-aged children. The fourth paper of this special issue presents the analysis of the prevalence of myopia among a sample of more than 6,000 children in Spain. The prevalence of myopia in Spain has increased from 17% in 2016 to 20% in 2017. Likewise, the number of children with high myopia has also increased, from 1.7% in 2016 to 3.6% in 2017. 43.3% of the participants spent more than 3 hours a day doing near activities, and 48.9% of this group spent more than 50% of this time using electronic devices. In addition, only 9.7% spent more than 2.5 hours outdoors each day. The incidence of myopia among schoolchildren in the experimental classes of the Air Force in China at the 3-year follow-up is 27.01%. A more hyperopic baseline refraction, more time spent outdoors, and longer writing/reading distance were protected against myopia onset, while more near-work time was a risk factor. Gender is associated with the prevalence of myopia among Polish schoolchildren ranging from 9 to 16 years of age. The prevalence of astigmatism decreased slightly over the two-year study period. Longer ALs and higher AL/CRC (axial length (AL) and corneal radius of curvature (CRC)) ratios were independent risk factors for developing CSA. Increased astigmatism was associated with AL growth, AL/CRC (axial length (AL) and corneal radius of curvature (CRC)) ratio increases, and the development of myopia. The eighth article presents the novel method of remotely monitoring and controlling the face device distance and illuminance that can potentially open new paths for myopia prevention and myopia control. The ninth paper evaluates the changes in retinal vessel density and thickness after small incision lenticule extraction (SMILE) with optical coherence tomography angiography (OCTA) in myopic patients. The vessel density at the parafoveal and perifoveal regions decreased at 1 day after SMILE with no effect on the visual acuity and relieved within 2 weeks. Decreased ocular blood flow in response to the spike in IOP may account for such changes. The tenth article addresses myopic anisometropia of more than 2D can that causes a significant impairment of binocular vision. Stereoacuity at distance is more sensitive to myopic anisometropia than stereoacuity at near. Myopic anisometropia involving “against the rule” astigmatism potentially affects binocularity more than anisometropia with regular astigmatism. A prompt correction of anisometropia of more than 2D is needed in children to prevent the development of amblyopia. The final paper describes the natural progression in Chinese patients with pathological myopia. Fundus degenerations in children with pathological myopia may lead its way since the age of 10 years. Besides, children with bilateral pathological myopia can have parallel development in visual acuity.

*Magorzata Mrugacz* *Marzena Gajecka* *Ewa Mrukwa-Kominek* *Katarzyna J. Witkowska*
